# The CCL5/CCR5 Axis in Cancer Progression

**DOI:** 10.3390/cancers12071765

**Published:** 2020-07-02

**Authors:** Donatella Aldinucci, Cinzia Borghese, Naike Casagrande

**Affiliations:** Molecular Oncology Unit, Centro di Riferimento Oncologico di Aviano (CRO), IRCCS, PN, I-33081 Aviano, Italy; cpborghese@cro.it (C.B.); naike.casagrande@cro.it (N.C.)

**Keywords:** cancer, CCL5, CCR5, tumor microenvironment, immunosuppression, CCR5 antagonist, drug resistance, metastasis

## Abstract

Tumor cells can “hijack” chemokine networks to support tumor progression. In this context, the C-C chemokine ligand 5/C-C chemokine receptor type 5 (CCL5/CCR5) axis is gaining increasing attention, since abnormal expression and activity of CCL5 and its receptor CCR5 have been found in hematological malignancies and solid tumors. Numerous preclinical in vitro and in vivo studies have shown a key role of the CCL5/CCR5 axis in cancer, and thus provided the rationale for clinical trials using the repurposed drug maraviroc, a CCR5 antagonist used to treat HIV/AIDS. This review summarizes current knowledge on the role of the CCL5/CCR5 axis in cancer. First, it describes the involvement of the CCL5/CCR5 axis in cancer progression, including autocrine and paracrine tumor growth, ECM (extracellular matrix) remodeling and migration, cancer stem cell expansion, DNA damage repair, metabolic reprogramming, and angiogenesis. Then, it focuses on individual hematological and solid tumors in which CCL5 and CCR5 have been studied preclinically. Finally, it discusses clinical trials of strategies to counteract the CCL5/CCR5 axis in different cancers using maraviroc or therapeutic monoclonal antibodies.

## 1. Introduction

In the tumor microenvironment, cancer cells destabilize the normal milieu of inflammatory cytokines and chemokines, and thereby exert tumor-promoting effects [1,2]. By secreting chemokines, cancer cells recruit T cells, monocytes, myeloid cells, fibroblasts and mesenchymal stromal cells (MSCs) from bone marrow or adipose tissue, and then “educate” [3] them to become immunosuppressive T regulatory cells (Tregs) [4], tumor-associated macrophages (M2-TAM) [5], myeloid-derived suppressor cells (MDSCs), [6], cancer-associated fibroblasts (CAFs) [7], and cancer-associated adipocytes [8], respectively. The result is the formation of a tumor-protecting microenvironment with anergic or exhausted T cells and natural killer (NK) cells [9]. In turn, tumor-educated normal cells support cancer cell growth, survival, migration and invasion, leading to metastasis [10]. Chemokines in distant organs also recruit tumor cells and facilitate their dissemination [11,12], while the paracrine activity of molecules released by adipocytes modifies antitumor immunity [13]. Moreover, tumor cells promote angiogenesis directly and indirectly by increasing the proangiogenic activity of TAMs [14]. Finally, the tumor microenvironment (TME) protects cancer cells from drug activity and reduces drug penetration by forming “sanctuaries” [15].

The presence of certain types of infiltrating inflammatory cells and the expression of a large number of inflammatory mediators (e.g., cytokines, chemokines, enzymes) are associated with poor prognosis and metastasis [2,16]. Thus, counteracting TME formation and interfering with chemokine-mediated effects are now considered an additional therapeutic approach [17]. Neoplastic tissues contain high levels of inflammatory mediators, including the C-C chemokine ligand 5 (CCL5) and the C-C chemokine receptor type 5 (CCR5) [1,18]. This review focuses on the CCL5/CCR5 axis, which is the main actor in tumor progression [19,20]. However, CCL5 is a double-edged sword in cancer, because it also promotes antitumor immunity by recruiting anti-tumor T cells and dendritic cells to the TME, and therefore enhances the immunotherapy response in different tumor types [21,22,23,24,25].

CCL5 (regulated upon activation, normal T cell expressed and secreted, Rantes) belongs to the C-C motif chemokine family whose members also include CCL3 (macrophage inflammatory protein 1-alpha, MIP-1α) and CCL4 (MIP-1β) [19,26]. CCL5 binds with high affinity to its receptor CCR5, but it also binds CCR1, CCR3, CCR4 [27,28], CD44 [27,29], and GPR75 [30]. CCL5 is a target gene of nuclear factor kappa-light-chain-enhancer of activated B cells (NF-κB) [28,31]. It is expressed by T lymphocytes, macrophages, platelets, synovial fibroblasts, tubular epithelium, and tumor cells [20].

CCR5 is a seven-transmembrane G protein-coupled receptor that mediates different signal transduction cascades in response to ligand binding. It is a promiscuous receptor that binds with high affinity to CCL5, CCL3, CCL4 and CCL8 (monocyte chemoattractant protein 2, MCP2). CCR5 engagement results in G protein activation and the activation of signal transduction cascades such as the protein kinase B (PKB, also known as Akt) and NF-kB pathways, cytoskeleton rearrangement and chemotactic cell migration [32]. CCR5 is expressed on various cell types including T cells, macrophages, dendritic cells, eosinophils and microglia [33]. It plays a fundamental role in the inflammatory response by directing cells to sites of inflammation [34]. It is the major co-receptor for the human immunodeficiency virus (HIV) type I [33,35]. Its interaction with CD34, a marker of hematopoietic stem cells, was found to promote cancer [36].

CCR5 overexpression can be the consequence of oncogenic transformation (Ha-Ras, c-Myc, ErbB2, and c-Src) [37]. In the TME, high levels of CCL5 or CCR5 can be the result of the education of normal cells [38] or the recruitment and accumulation of CCR5+ cells (lymphocytes, monocytes, MSCs, adipocytes). CCL5 and/or CCR5 overexpression has been found in many tumors, including acute lymphocytic leukemia [39], Hodgkin lymphoma [38,40], multiple myeloma [41,42], breast cancer [37,43], colorectal carcinoma [44,45], esophageal cancer [46], gastric adenocarcinoma [47,48], head and neck cancer [49], melanoma [50], pancreatic cancer [51], and prostate cancer [52].

## 2. Involvement of the CCL5/CCR5 Axis in Cancer Progression

**Figure 1:**
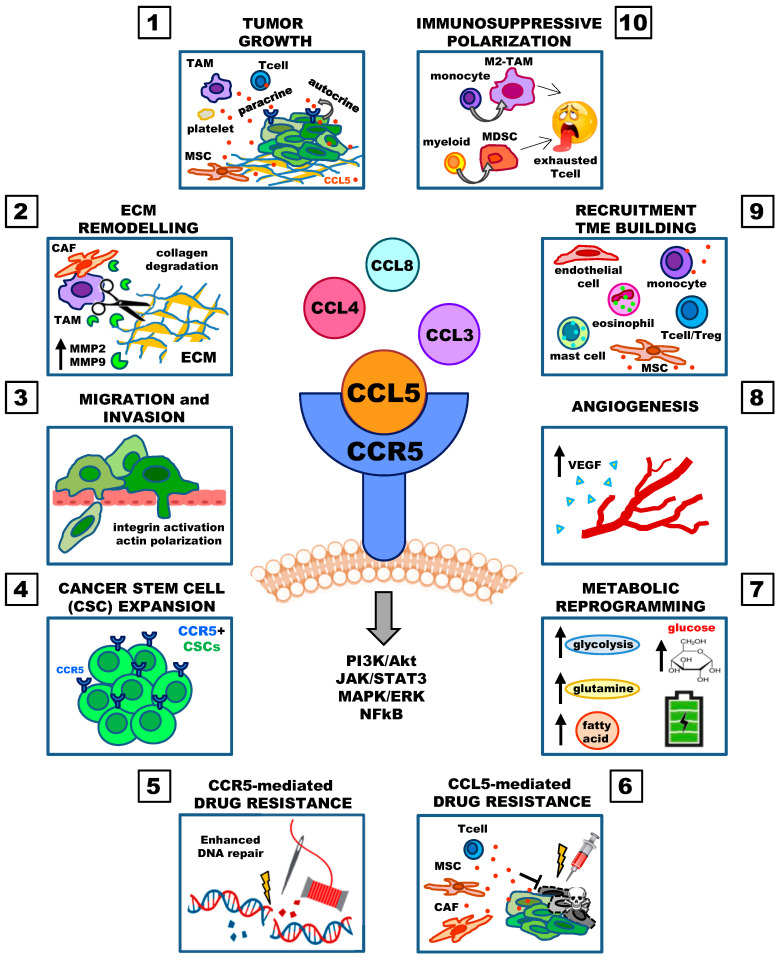
Involvement of the CCL5/CCR5 axis in cancer progression. (**1**) CCL5, secreted by tumor cells (autocrine) and by the TME (paracrine), increases tumor growth. (**2**) CCL5 induces collagen degradation by activating matrix metalloproteinases in macrophages and fibroblasts. (**3**) CCL5 increases tumor cell migration and invasion by integrin activation (adhesion) and actin polarization. (**4**) CCR5+ cancer cells show characteristics of cancer stem cells. (**5**) CCR5 expression associates with stronger repair responses to DNA damage induced by doxorubicin and γ-irradiation. (**6**) CCL5 secreted by tumor cells and by the TME decreases drug cytotoxic activity. (**7**) CCR5 engagement stimulates glucose uptake, glycolysis, pentose phosphate pathway, fatty acid synthesis, and glutamine metabolism. (**8**) CCL5 promotes endothelial cell migration and neovessel formation, and induces the secretion of VEGF by endothelial cells. (**9**) CCL5 secreted by tumor cells recruits normal cells to build the TME. (**10**) CCL5 induces the immunosuppressive polarization of monocytes and myeloid cells leading to M2-TAMs and MDSCs that induce exhaustion of effector T-cells. Protein kinase B, Akt; cancer-associated fibroblast, CAF; C-C chemokine ligand 5, CCL5; C-C chemokine receptor type 5, CCR5; cancer stem cell, CSC; extracellular matrix, ECM; extracellular signal-regulated kinase, ERK; janus kinase, JAK; mitogen-activated protein kinase, MAPK; matrix metalloproteinase, MMP; myeloid-derived suppressor cell, MDSC; mesenchymal stem cell, MSC; nuclear factor kappa-light-chain-enhancer of activated B cells, NF-kB; phosphoinositide-3-kinase, PI3K; signal transducer and activator of transcription protein, STAT3; tumor-associated macrophage, TAM; tumor microenvironment, TME; regulatory T cell, Treg; vascular endothelial growth factor, VEGF.

The CCL5/CCR5 axis facilitates tumor progression through multiple mechanisms (Figure 1). It increases tumor growth (1), induces extracellular matrix remodeling (2), enhances tumor cell migration (metastasis formation) (3), supports cancer stem cell expansion (4), enables cancer cell resistance to drugs (5,6), decreases the cytotoxicity of DNA-damaging agents (5), deregulates cellular energetics (metabolic reprogramming) (7), promotes angiogenesis (8), recruits immune and stromal cells (9), and induces the immunosuppressive polarization of macrophages (10).

### 2.1. Tumor Growth

CCR5 engagement increases tumor growth in both autocrine and paracrine manners. Autocrine growth is characterized by the presence of functional CCR5 and the secretion of its ligands by cancer cells [40], while paracrine growth is induced by CCR5 ligands secreted by normal cells of the TME like T lymphocytes, macrophages, fibroblasts, MSCs and CAFs [38,52]. CCR5 ligands stimulate cell growth by inducing the mammalian target of rapamycin (mTOR) pathway followed by a rapid up-regulation of cyclin D1, c-Myc and Dad-1 expression [53], by activating the Jak–STAT pathway [54], and by enhancing glucose uptake with increased ATP production and glycolysis associated with extracellular acidification [55].

### 2.2. Extracellular Matrix Remodeling and Migration

Binding of CCL5 to its receptors activates a series of downstream effects that facilitate receptor internalization and signal transduction, leading to activation of integrins (adhesion) and polarization of actin cytoskeleton [32]. CCL5 activates αvβ3 integrin, leading to cell migration through PI3K/Akt, which in turn activates inhibitor of nuclear factor-κB (IκB) kinase (IKK) alpha/beta and NF-κB [32,56,57]. CCL5 induces collagen degradation by activating matrix metalloproteinase 1 (MMP-1) and MMP-13 expression in human rheumatoid arthritis synovial fibroblasts [58]. CCL5 induces MMP-9 secretion and collagen degradation by monocytes, supporting tumor cell extravasation [59]. In prostate cancer, CCL5 promotes invasion by increasing the secretion of both MMP-2 and -9 and by activating extracellular signal-regulated kinases (ERK) and Rac signaling [60]. In osteosarcoma, CCL5/CCR5 interactions act via MEK and ERK, and activate NF-κB, resulting in the activation of αvβ3 integrin [57]. In breast cancer, CCL5 secreted by MSCs of the TME increases tumor cell motility and invasion [10] or recruits tumor cells in distant organs, facilitating their dissemination [11].

### 2.3. Cancer Stem Cell Expansion, Decreased Cytotoxicity of DNA-Damaging Agents, Drug Resistance

The expression of CCR5 also determines the fate of cancer cells in the absence of ligands [43]. CCR5+ breast cancer cells show features of cancer stem cells: in mice, they form mammospheres and tumors with greater efficiency than CCR5− tumor cells. CCR5 expression associates with stronger repair responses to DNA damage by DNA-damaging agents (doxorubicin and γ-irradiation) [43]. CCR5 inhibitors enhance both doxorubicin and γ-irradiation cytoxicity. Single-cell analysis of breast cancer cells showed that CCR5 controls cell survival signaling pathways [43]. In breast, ovarian and prostate cancer, CCL5 secreted by the TME can reduce drug activity. CCL5 secreted by T cells decreases chemotherapy activity in prostate cancer [61]. Cisplatin, by inducing the secretion of CCL5 by CAFs, decreases its activity in ovarian cancer [62]. Autocrine CCL5 reduces the sensitivity to tamoxifen of breast cancer cells [63].

### 2.4. Deregulated Cellular Energetics (Metabolic Reprogramming)

Reprogramming of cancer metabolism is a newly recognized hallmark of malignancy [64]. The aberrant glucose metabolism in cancer cells associates with cell proliferation and resistance to radiation and chemotherapy. Tumor cells require higher rates of glucose uptake, and have higher catabolite uptake and use [65,66]. In breast cancer, CCR5 engagement induces Akt phosphorylation, stimulating glucose uptake, glycolysis, the pentose phosphate pathway, fatty acid synthesis, and glutamine metabolism [55].

### 2.5. Angiogenesis

CCL5 supports proangiogenic activity by increasing the migration of endothelial cells, spreading, neovessel formation, and the secretion of vascular endothelial growth factor (VEGF) [67]. Tumor cells, upon CCL5 stimulation, produce VEGF [68,69]. By secreting CCL5, tumor cells recruit CCR5+ monocytes/macrophages [11,26], which induce angiogenesis through VEGF secretion [19,68,70]. Thus, counteracting the recruitment of TAMs, which are now considered to be the major player in the regulation of tumor angiogenesis, may be an effective therapeutic strategy. CCL5 can promote lymphangiogenesis [69].

### 2.6. Recruitment of Immune and Stromal Cells and Immunosuppressive Polarization

Tumor cells, by secreting CCL5, recruit normal cells and then take control of (educate) them [38]. Recruited monocytes are educated to become protumoral immunosuppressive M2-TAMs [38]. M2-TAMs express programmed death-ligand 1 (PD-L1), CD206, and indoleamine 2,3-dioxygenase, and produce anti-inflammatory cytokines such as interleukin (IL) 10 and transforming growth factor β (TGF-β), which expand immunosuppressive Tregs [3]. CCL5 recruits T cells and CCR5+ Tregs [38]. Tregs are immunosuppressive CD4+ T cells that control not only effector T cells but also B cells, NK cells, dendritic cells and macrophages via humoral and cell-cell contact mechanisms [71]. Tregs have inhibitory effects on tumoricidal lymphocytes that become anergic or exhausted T cells [70]. Both fibroblasts and MSCs are recruited by CCR5 ligands secreted by cancer cells, and are induced to secrete CCL5 and to become tumor-promoting CAFs [72,73]. CAFs support cancer cell proliferation, migration, metastasis formation [74] and epithelial–mesenchymal transition (EMT) [75]. CAFs promote drug resistance, recruit monocytes and Tregs, inhibit effector T cells and activate Treg functions [76]. Tumor-educated MSCs are immunosuppressive and tumor protective [77] and, by secreting CCL5, they recruit CCR5+ monocytes [38]. The CCL5/CCR5 axis is involved in immunosuppressive TAM polarization in cancer tissues [44]. In the bone marrow, CCL5-overexpressing hemopoietic cells become MDSCs, a heterogeneous myeloid cell population that limits productive immune responses against tumors [78].

## 3. The Tumor-Promoting Role of the CCL5/CCR5 Axis

To study the CCL5/CCR5 axis in cancer, different approaches have been used: inhibition of CCR5 with antagonists, inhibition of CCL5 expression with neutralizing antibodies or gene silencing, and generation of CCL5-knockout mice. The CCR5 antagonists used in preclinical studies are maraviroc, vicriviroc, TAK-779, anibamine, and BMS-813160.

Maraviroc is the most used CCR5 antagonist. It is an antiretroviral compound approved for the treatment of patients with HIV/AIDS, since CCR5 is a co-receptor for HIV-1 entry to host cells and is a target of anti-HIV therapies [17]. Maraviroc interferes with the binding of CCL5 to CCR5 and counteracts HIV entry into T cells and macrophages [17,79]. It binds the active site pocket of CCR5, thereby inactivating it. Maraviroc is a substrate for CYP3A4 and P-glycoprotein (multidrug resistance protein 1, MDR1), which reduces drug uptake or causes drug efflux out of cancer cells and is involved in drug resistance. The transport and absorption in the intestine and bile is regulated by MDR1 [80]. It is metabolized by cytochrome P450 3A4.

Vicriviroc is a piperazine-based CCR5 antagonist with activity against HIV. Vicriviroc was designed to bind CCR5 and inhibit the entry of HIV into CD4 cells. TAK-779 is a non-peptide antagonist of CCR5, CCR2b and CXCR3 [79]. Anibamine, the first natural CCR5 antagonist, is a unique pyridine quaternary alkaloid, recently isolated from *Aniba panurensis*; it has moderate binding affinity to CCR5 [81,82]. BMS-813160 is an antagonist of both CCR2 and CCR5, with potential immunomodulating and antineoplastic activities [17].

### 3.1. The CCL5/CCR5 Axis in Hematological Malignancies

CCR5 contributes to tumor progression in several hematological cancers, including acute lymphoblastic leukemia [39], acute myeloid leukemia, chronic myeloid leukemia [83], classic Hodgkin lymphoma [38], and multiple myeloma [84]. The effects of disrupting the CCL5/CCR5 axis in hematological malignancies using antibodies or antagonists are discussed in the following paragraphs and summarized in Table 1.

#### 3.1.1. Acute Myeloid Leukemia

The CCL5/CCR5 axis exerts profound effects on the progression of acute myeloid leukemia (AML) [87]. AML cells express CCR5 and its ligands [87,88]. AML patients are classified into subsets according to their chemokine responsiveness and chemokine expression profile [89]. AML patients with the monocytic phenotype have high serum levels of CCL5, CCL4, and CCL3 [90]. After the first stage of chemotherapy in these patients, CCR5 expression decreases and CCL3 returns to baseline levels, whereas CCL5 and CCL4 only decreases [90].

CCL5 is involved in resistance to inhibitors of fms-like tyrosine kinase 3 (FLT3) [91]. FLT3-internal tandem duplication (ITD)-mutated AML cell lines resistant to midostaurin, a tyrosine kinase inhibitor approved for the treatment of FLT3-mutant AML, secrete high levels of CCL5. Consistently, in AML cells, CCL5 overexpression induces midostaurin resistance by the up-regulation of survival/proliferation pathways [91]. FLT3-ITD-mutated AML blasts and midostaurin-relapsed AML patients have high levels of CCL5, suggesting CCL5 as a biomarker for predicting resistance to tyrosine kinase inhibitors in AML [91].

A high number of Tregs were found in peripheral blood and bone marrow of newly diagnosed and relapsed/refractory AML patients, compared with healthy controls [92]. Blocking Treg migration to the TME with maraviroc prolonged survival in a mouse leukemia model and enhanced the antileukemic effects of CD8+ T cells [85].

#### 3.1.2. Acute Lymphoblastic Leukemia and Chronic Lymphoblastic Leukemia

Acute lymphoblastic leukemia (ALL) originates from hematopoietic precursors committed to a B or T cell lineage, and is the most common hematological malignancy diagnosed in children [93]. ALL cells overexpress CCR5 [39]. In vitro, maraviroc inhibited ALL cell growth, induced apoptosis, decreased migration to C-X-C motif chemokine ligand 12 (CXCL12) and CXCL13, decreased adhesion to fibronectin and vascular cell adhesion molecule 1, and down-regulated Jak/STAT signaling. Moreover, maraviroc suppressed ALL xenograft growth [39].

CCL3 is secreted by cultured chronic lymphoblastic leukemia (CLL) cells, especially those expressing the bad prognosticators CD38 and CD49d [94]. CCL3 was found in CLL cell cytoplasm from bone marrow biopsies [94]. CCL3 increases monocyte migration, and CD68+ macrophage infiltration is particularly high in bone marrow of CD38+CD49d+ CLL patients [95].

#### 3.1.3. Hodgkin Lymphoma

Classic Hodgkin lymphoma (cHL) cells, which account for less than 1%, need the support of the TME to grow and survive [96]. The TME is composed of a vast majority of normal cells that, to become protumorigenic, have to be educated by tumor cells [96]. In this context, CCL5 plays a fundamental role in TME building [96] and tumor growth [3]. CCL5 and CCR5 are constitutively expressed by cHL-derived cell lines [40,97] and by tumor cells from cHL tissues [38,40]. CCR5 is expressed by stromal cells, macrophages and lymphocytes of the cHL TME [38,98]. Both CCL5 and CCL3 are higher in Epstein-Barr virus (EBV)-positive than in EBV-negative HL tissues [99], consistent with the fact that the EBV gene LMP1 induces the expression of CCL5 in EBV-negative cell lines [100].

CCR5 in cHL cells is fully functional and its ligands function as paracrine [38] and autocrine [40] growth factors. CCL5 secreted by cHL cells increases the migration of mast cells [97], eosinophils, CD4+ T cells [40], monocytes [38], MSCs [38] and likely Tregs [101], highlighting its involvement in TME formation [102].

CCL5 secretion is increased by CD40 engagement in cHL cells [31] or by their coculture with MSCs from HL-involved lymph nodes [40]. The silencing of interferon regulatory factor 4 (IRF4), a transcription factor overexpressed by cHL cells [103] and involved in cancer cell proliferation and survival, decreases CCL5 secretion [104]. Conditioned medium from cHL cells increases CCL5 secretion by bone marrow MSCs (tumor-educated MSCs) [38]. High levels of CCL5 in cHL tissues correlate with poor prognosis and monocyte infiltration [38]. Maraviroc decreases both MSC and monocyte recruitment by cHL cells and monocyte recruitment by CCL5-secreting tumor-educated bone marrow MSCs. Maraviroc slightly decreases tumor cell growth and inhibits growth induced by CCR5 ligands secreted by monocytes and MSCs. It enhances the cytotoxic activities of doxorubicin and brentuximab vedotin [38]. In a heterospheroid model of TME interactions, generated by the three-dimensional coculture of cHL cells with HL-MSCs and monocytes, maraviroc counteracts heterospheroid formation and decreases cell viability [38]. In mice bearing cHL tumor xenografts, maraviroc reduces tumor growth by more than 50% and inhibits monocyte accumulation [38].

#### 3.1.4. Multiple Myeloma

CCR5 and CCR1 are expressed by multiple myeloma (MM) cells and their engagement induces tumor cell survival, migration and homing to the bone marrow [41,86,105,106]. CCL5 increases MM cell migration that correlates with CCR5 expression levels [41,105]. MM cells localize in and interact with the bone marrow, leading to microenvironment alterations that are critical for tumor progression, therapy resistance, and osteolytic bone destruction [107]. They promote osteoclast formation, which in turn enhances tumor cell proliferation via cell–cell contact [108].

CCR5 has an essential role in bone-destructive conditions through the functional regulation of osteoclasts [109]. One of the most important osteoclast-activating factors produced by MM cells is the CCR5 ligand CCL3 [108], which also inhibits osteoblast formation, further increasing bone resorption [108,110]. In MM patients, high serum levels of CCL3 (and CCL4) positively correlate with the extent of bone disease, bone resorption, and poor prognosis [108,111]. High levels of CCL3 in bone biopsies correlate with extensive bone disease, increased angiogenesis and advanced stage in newly diagnosed MM patients [112]. Indeed, MM cells from patients with multiple bone lesions secrete high amounts of CCL3 [108] and, in accordance, osteolytic lesions correlate with the production of both CCL3 and CCL4 by MM cells [113]. Inhibition of CCR1 and CCR5 by antagonists or neutralizing antibodies partially reduces osteoclastogenesis, osteolytic lesions and MM-induced angiogenesis [41,84,86,114].

Overall, CCR5 and its ligands are involved in MM progression by promoting tumor growth, recruitment of tumor cells to the bone marrow, osteoclast activation, and osteoblast inhibition, leading to bone destruction [41].

#### 3.1.5. Other Lymphomas

In patients with extranodal NK/T-cell lymphoma (ENKTL), overall survival was lower in patients with measurable CCL3 serum levels than in those with undetectable levels [115]. In vitro studies demonstrated the expression of CCR1 and CCR5 on the surface of patient-derived ENKTL cells and the positive effect of CCL3 on their growth [115].

The expression of CCR5 was evaluated in lymph nodes derived from B-cell non-Hodgkin lymphoma (NHL) and in reactive lymphatic tissues (reactive lymph nodes) [116]. Lymph nodes, peripheral blood, and bone marrow aspirates from patients were taken at diagnosis and after completed chemotherapy. Reactive lymph nodes expressed low levels of CCR5, but higher CCR5 gene expression in samples from lymphoma patients correlated with advanced stage of the disease, high proliferation index (Ki-67), and international prognostic index. Patients with higher CCR5 expression had shorter survival, suggesting a possible involvement of CCR5 in NHL progression [116].

### 3.2. The CCL5/CCR5 Axis in Solid Tumors

CCL5 or CCR5 is expressed in various solid tumors. Here we summarize recent knowledge of the actions of these proteins in breast cancer, breast phyllodes, cholangiocarcinoma, colorectal cancer, esophageal squamous cell carcinoma, gastric cancer, glioblastoma, head and neck cancers, lung cancer, melanoma, osteosarcoma, and ovarian, pancreatic, pituitary, prostate and thyroid cancers. In Table 2, we summarize the effects of disrupting the CCL5/CCR5 axis in solid tumors.

#### 3.2.1. Breast Cancer

CCL5 expression is rare in epithelial cells of normal ducts or of benign breast lumps, but is acquired during malignant transformation [26]. CCL5 is secreted by breast cancer cells [37], leukocyte infiltrates near tumors [37], and by non-malignant stromal cells at the primary tumor site or sites of metastasis [10,146,147]. CCL5 has been detected in primary tumors and in metastases (regional lymph nodes). Its expression is high in advanced disease stages (stages II and III), especially in triple-negative breast cancer [148], and low in benign breast disorders and in women who have had reduction mammoplasty [37]. Serum CCL5 is higher in breast cancer patients than in healthy individuals [37] and tends to be higher in advanced disease stages [148].

CCL5, in association with the negativity for estrogen receptor (ER), was suggested as a biomarker for disease progression in stage II breast cancer patients [37]. High CCL5 levels in serum and patient samples positively correlate with lower rates of complete response after neoadjuvant therapy [120]. In patients with early HER2-positive breast cancer, CCL5 levels correlate with ERK phosphorylation, worse disease-free survival, and overall survival. By silencing CCL5 and by using maraviroc, it was demonstrated that CCL5 overexpression is linked to acquired resistance to trastuzumab and that this phenomenon is mediated by ERK activation [120].

CCR5 expression is higher in breast cancer tissues than normal tissues [37]. A microarray analysis on more than 2000 human breast cancer samples revealed high CCR5 levels in the basal and HER-2 genetic subtypes [37]. In breast cancer cell lines, CCR5, when expressed, correlates with increased invasive properties [37,149], and increased CCR5 levels correlates with increased migratory abilities [37]. CCR5 contributes to certain characteristics of breast cancer stem cells [43]. CCR5+ breast cancer cells have increased ability to form mammospheres and tumors in mice, and present enriched expression of pathways mediating DNA repair with respect to CCR5− cells [43]. Functional analysis demonstrated that endogenous CCR5 enhances DNA repair gene levels (homology directed repair and single strand annealing) in response to DNA damaging agents. Accordingly, maraviroc and vicriviroc increase cancer cell killing mediated by the DNA-damaging agent doxorubicin and by high dose γ-radiation [43]. Under hypoxic conditions, breast cancer cells strongly increase CCR5 and CCL5 expression, thereby stimulating cancer cell migration [150]. Moreover, hypoxia-inducible factor 1α expression correlates with CCL5 and CCR5 levels in clinical samples [150].

CCL5 mediates the cross-talk between breast cancer cells and MSCs in the TME [10,147]. Cancer cells stimulate CCL5 secretion by MSCs, and MSC-derived CCL5 promotes tumor cell invasion [10] and MMP activation [151]. In vivo studies demonstrate the involvement of the CCL5/CCR5 axis in lung metastasis and colonization after the injection of breast cancer cells and MSCs in mice [10]. CCL5 secreted by hematopoietic cells is involved in breast cancer progression [78,152]. Scarcity of CCL5 secreted by hematopoietic precursors, rather than that from peripheral tissues or tumor cells, reduces primary tumor growth and metastatic disease in mice. Hematopoietic CCL5 supports the generation of MDSCs capable of suppressing CD8+ cytotoxic T cells [78,152]. Silencing host CCL5 in bone marrow, in combination with maraviroc, had robust anti-tumor immunity effects and great therapeutic efficacy against breast cancer xenografts [78].

In breast cancer, a population of residual tumor cells can survive after treatment and persist in a dormant state for many years. Residual Her2-driven breast tumors, responsible for cancer recurrence, are characterized by a pro-inflammatory gene expression program, including CCL5 [153]. High levels of CCL5 decreased the time between tumor treatment and recurrence [153]. CCL5 was found to promote tumor recurrence by recruiting CCR5-expressing macrophages, which contribute to collagen deposition in residual tumors. Increased collagen deposition was suggested to promote tumor growth since high levels of collagen in patients were found in recurrent tumors and correlated with worse outcomes [153].

Orthotopically implanted metastatic mammary tumors induce Treg accumulation in the lungs, a typical site of mammary tumor metastasis [117]. Tregs in the primary tumor and in lung metastases express higher levels of CCR5 than Tregs in tumor-free mice. Tregs in metastatic lungs express higher levels of CCR5 than do other immune cell populations. CCL8, another CCR5 ligand, is produced by macrophages in the lungs of mice with metastases. Maraviroc reduced Treg migration toward CCL8, and in vivo it reduced CCR5(+) Tregs and metastatic tumor burden in the lungs without affecting primary tumor growth [117].

CCR5 is required for bone marrow-derived endothelial progenitor cell-mediated angiogenesis [118]. In vitro, CCR5 expression increased in endothelial cells in response to breast cancer conditioned medium, and its levels correlated with breast cancer invasive grade. In a syngenic mouse model of breast cancer, maraviroc reduced endothelial cell migration and angiogenesis, leading to impaired tumor growth, less metastasis, and improved survival [118].

Cross-talk between triple-negative breast cancer cells and stromal lymphatic endothelial cells (LECs) is involved in tumor growth and metastasis [154]. Tumor cells secrete IL-6, which interacts with the IL-6 receptor (IL-6R) on tumor-associated LECs in TME or on LECs in distant organs. The engagement of IL-6R stimulates the STAT3 signaling pathway, resulting in the up-regulation of CCL5 expression and secretion by LECs. In pre-metastatic organs, CCL5 formed a chemotactic gradient to recruit CCR5-positive cancer cells and thus facilitated tumor cell dissemination [119]. In breast cancer tumor xenografts, the combination of maraviroc and cMR16-1 (anti-murine IL-6R antibody) dramatically inhibited tumor growth and prevented thoracic metastasis, compared to the single agents [119]. Maraviroc and tocilizumab (anti-IL-6R antibody) showed high synergism in triple-negative breast cancer cell viability and was proposed as a therapeutic option to block metastasis through the lymphatic system [119].

#### 3.2.2. Breast Phyllodes

Breast phyllodes are rare tumors that start in the connective (stromal) tissue of the breast [155]. Nie et al. [122] reported that breast phyllode tumor cells recruit and polarize TAMs towards an M2-like protumorigenic phenotype by secreting CCL5. In a murine patient-derived xenograft model of malignant phyllodes, maraviroc, by counteracting the cross-talk between tumor cells and TAMs, prevented monocyte recruitment to the tumor, and therefore reduced tumor growth; it was more active than doxorubicin. Given the lack of chemotherapy regimens for these tumors, maraviroc may be a new treatment for breast phyllodes to counteract the protumorigenic activity of TAMs [122].

#### 3.2.3. Cholangiocarcinoma

In cholangiocarcinoma (bile duct cancer), the presence of TNF-α and IFN-γ, due to early inflammatory, stimulates MSCs to secrete TNF-α, CCL5 and IL-6, to express indoleamine 2,3-dioxygenase, to activate NF-κB, and to increase their migratory abilities [123]. In turn, stimulated MSCs enhance the expression by cholangiocarcinoma tumor cell lines of CCR5, MMP2 and MMP9, migration, and metastasis formation in tumor xenografts. Maraviroc and CCL5-neutralizing antibodies inhibited MSC-induced migration of tumor cells in vitro [123].

#### 3.2.4. Colorectal Cancer

CCR5 expression was detected within the cytoplasm of several colorectal cancer (CRC) cell lines [126]. CCR5 is absent on tumor cells in the early stages of CRC but is present in T cells [156]. CCR5 and CCL5 are overexpressed in CRC cells of primary tumors and of metastases to liver and lung [126]. CCR5 expression correlates with CRC prognosis [126]. Stage III/IV patients with CCR5-high CRC had poorer prognosis than those with CCR5-low. [126]. In blood, CCR5 and CCL5 levels were higher in CRC patients than healthy controls [157]. Together with platelet-derived growth factor (PDGF-BB) and ephrinA7 (EphA7), CCR5 and CCL5 are potential biomarkers for the early diagnosis of CRC [157].

In vitro, CCL5 increased the growth and migratory response of CRC cells. In tumor xenografts, inhibition of CCL5 with neutralizing anti-CCL5 antibodies decreased growth, liver metastasis and peritoneal carcinosis. CCR5 targeting with TAK-779 slightly reduced CRC growth [126].

In CRC, the CCL5/CCR5 axis is involved in the formation of an immunosuppressive TME. Chang et al. [4] found that high levels of CCL5 expression in human CRC tissues correlated with increased infiltration of Tregs and apoptotic CD8+ T cells. CCL5 promoted Treg migration into tumors and enhanced apoptosis of CD8+ T cells, which was associated with the increased release of TGF-β by Tregs [4]. In mouse models of CRC, the knockdown of tumor cell CCL5 and the blockade of CCR5 signaling decreased Treg infiltration and apoptosis of tumor-infiltrating CD8+ T cells and reduced tumor growth [4].

Zhang et al. [128] found that CCL5-deficient mice, via facilitating the intratumoral accumulation of CD8+ T cell, delayed CRC tumor growth and metastasis formation. This is likely due to the decreased secretion by TAMs of S100a9, a calcium-binding protein considered a pro-inflammatory mediator involved in CD8+ T cell migration into the low hypoxic area of tumor tissues. CRC patients with a low number of infiltrating CD8+ T cells into tumor tissues expressed high levels of both CCL5 and S100a9. CCL5 levels correlated negatively with the number of intratumoral CD8+ T cells but positively with S100a9 expression [128]. These results suggest a possible therapeutic strategy with checkpoint inhibitors, since CCL5 deficiency in the mouse CRC model up-regulated the expression of programmed cell death protein 1 (PD-1) and programmed death-ligand 1 (PD-L1), and reduced the resistance to anti-PD-1 antibody [128].

In the late phase of colitis-associated colon carcinogenesis, CCR5+ CAFs, recruited by CCL3, accumulate in the TME [158]. Tanabe et al. [124] demonstrated that maraviroc, by counteracting the accumulation of CAFs in tumor tissues, reduced the orthotopic in vivo growth of CRC cells. Maraviroc had few effects on leukocyte infiltration, but reduced the number of α-smooth muscle actin-positive CAFs in the TME. Since CAFs secrete epidermal growth factor, a growth factor for colon cancer cells, the reduced growth could be attributed to a decreased proliferating stimulus [124].

Halama et al. [44] studied the role of the CCL5/CCR5 axis in CRC in a human ex vivo metastasis explant model (organoid) and in a pilot clinical study (MARACON, ClinicalTrials.gov identifier, NCT01736813) of advanced CRC [44]. They found that at the invasive margins of metastatic CRC, T cells, attracted by chemokines released by CD68+ and CD11b+ myeloid cells, secrete CCL5. CCL5 stimulated tumor growth and invasiveness and promoted immunosuppressive M2-TAM polarization [44]. In organoids obtained from patients with metastatic CRC, maraviroc repolarized macrophages towards an anti-tumoral M1-like state [44]. In the MARACON trial, 14 patients with advanced CRC refractory to standard chemotherapy received maraviroc daily for 2 months. The treatment induced objective partial responses in three CRC patients with liver metastases, without major side effects [44].

The same authors performed a systematic analysis of CCR5 expression by cancer cells in immunohistochemistry tissue samples from five different cohorts (*n* = 97 specimens), including the MARACON trial cohort [159]. Materials were analyzed for CCR5 expression (using immunohistochemistry), CCR5 delta 32 mutation (polymerase chain reaction, PCR), immune cell distribution, density and activation, tumor cell death, cytokine and chemokine patterns [159]. CCR5 expression was found to increase with primary tumor size and peaks in T4 CRC tumors (metastatic colon cancer). In liver metastases, CCR5 intensity increased, compared to primary tumors, but the stain was detected in small isolated areas (patchy staining). Low CCR5 expression in metastases was found to characterize patients with prolonged disease-free survival and disease-specific survival. Patchy CCR5 expression in cancer cells is a signature of liver metastases, and maraviroc was still effective in patients with CCR5 “patchiness”. Patchy CCR5 expression was found associated with an immunosuppressive TME, characterized by a low cytotoxic-to-regulatory T cell ratio at the invasive margin, and increased markers of M2-TAM (immunosuppressive polarization). Higher numbers of PD-1- and CTLA-4-positive cells surrounded tumors with patchy CCR5 expression, suggesting that this type of tumor could respond to immune checkpoint blockade [159]. Another clinical trial (PICCASSO; ClinicalTrials.gov identifier, NCT03274804), combining pembrolizumab (anti-PD-1) and maraviroc in previously treated subjects who have refractory microsatellite stable metastatic CRC was completed in March 2020.

In CRC, CCL5 secreted by TAMs facilitates immune escape [127]. Macrophage infiltration, induced by lipopolysaccharide or a high-cholesterol diet, promotes CRC growth, and macrophage-derived CCL5 inhibits cytotoxic T cell antitumor activity. CCL5 stabilizes PD-L1 expression in cancer cells due to the up-regulation of COP9 signalosome 5 (CSN5), a modulator of PD-L1 deubiquitination which has been associated with significantly shorter survival [127].

MSCs produce abundant CCL3, CCL4 and CCL5 [125]. The co-injection of MSCs and CCR5-overexpressing tumor cells promoted in vivo tumor xenograft growth. The tumor-promoting ability of MSCs was abolished by maraviroc, confirming the importance of CCR5 signaling in the cross-talk between MSCs and CRC cells [125].

#### 3.2.5. Esophageal Squamous Cell Carcinoma

Esophageal squamous cell carcinoma cells derived from metastatic lymph nodes produce higher levels of CCL5 than those from primary lesions and express both CCR3 and CCR5 receptors, while low levels or absence of chemokine and chemokine receptors are detected in normal esophageal epithelial cells [46]. CCL5 knockdown by small interfering RNA (siRNA) reduces cancer cell growth, migration and invasiveness and induces apoptosis. Maraviroc blocks esophageal squamous cell carcinoma cell migration and invasion in vitro, but not tumor growth [46].

#### 3.2.6. Gastric Cancer

The CCR5/CCL5 axis plays a crucial role in gastric cancer (GC) progression [47]. Higher serum CCL5 levels were detected in GC patients than in healthy people [54,160] and positively correlated with disease stage, shorter survival and poor prognosis [161]. These patients have strong CCL5 immunohistochemistry staining in tumor tissues [54,160] and in metastatic lymph nodes [162]. Highly metastatic GC cell lines secrete high levels of CCL5 [163].

CCR5 is expressed by GC cell lines [164,165]. In human GC tissue, CCR5 is associated with lymph node metastasis and worse prognosis [162,166]. Conditioned medium from highly metastatic GC cell lines enhances CCL5 expression in peripheral blood mononuclear cells (PBMCs). In turn, PBMCs increase GC cell invasion properties, which are reduced by neutralizing anti-CCL5 antibodies [129].

CD4+ tumor-associated lymphocytes express CCL5, and coculture with GC cells further increases CCL5 secretion by CD4+ T cells [130]. CCL5 enhances GC cell line growth. CCL5-treated GC cells cocultured with PBMCs induce apoptosis of CD8+ T cells via the Fas/Fas ligand pathway, but not of CD4+ T cells. An anti-CCL5 neutralizing antibody reduced tumor xenograft growth of GC cells coinjected with PBMCs [130].

In GC, CAFs expressing KLF5 (DNA-binding transcriptional regulator Krüppel-like factor) increase GC cell proliferation, migration, and invasion by activating the CCL5/CCR5 axis [167]. Accordingly, down-regulation of KLF5 inhibits the tumor-promoting effects of CAFs, which are restored by exogenous addition of CCL5. Overexpression of KLF5 by stromal cells, which is more frequently observed in CAFs than in normal fibroblasts, is associated with poor prognosis in GC [167].

In GC tissues, CCL5 levels correlate with the macrophage marker CD68 [168]. Tumor size, degree of tumor invasion, lymphatic metastasis and pathological grading correlate with high levels of CD68 and CCL5 [54]. The coculture of THP-1-derived macrophages with GC cells up-regulated CCL5, MMP2, and MMP9 in THP-1 cells, and increased tumor cell proliferation, clonogenic growth and migration [54]. Ding et al. [54] suggested that CCL5 secreted by TAMs, by activating the STAT3 signaling pathway, mediates gastric tumorigenesis and that CCL5 may be a therapeutic target for GC.

#### 3.2.7. Glioblastoma

Glioblastoma cells secrete CCL5 and express CCR5, which is also expressed by stromal cells of the TME [169]. CCL5 is an autocrine growth factor and the CCL5/CCR5 axis mediates both monocyte and MSC infiltration in the TME.

In patients with glioblastoma multiforme (GBM), high CCR5 expression associates with poor prognosis [132]. CCL5 mediates activation of Akt, and induces proliferation and invasive responses in U87 and U251 GBM cell lines. Down-modulation of CCR5 decreases U87 tumor xenograft growth. Consistently, CCR5 expression positively correlates with increased p-Akt expression in GBM samples from patients [132].

TAMs are abundant in glioblastoma and facilitate growth and invasion of tumor cells. Hypoxia and macrophage infiltration in tumor tissues promote GBM invasion, and hypoxia up-regulates CCL4 in macrophages and CCR5 in cancer cells [170]. Conditioned medium from hypoxic macrophages has higher concentrations of CCL4, promotes MMP-9 expression, and has greater pro-invasion activity than conditioned medium from macrophages cultured in normoxic conditions [170].

CCL5 produced by low-grade tumor-associated microglia of the glioblastoma TME is responsible for maintaining neurofibromatosis type 1 (NF1) mouse optic glioma growth in vivo [171]. CCL5 expressed by mesenchymal GBM induces survival in an autocrine manner [172]. CCL5 expression is increased in mesenchymal GBM, a subtype of GBM characterized by the loss of the NF1 gene. CCL5 silencing reduces mesenchymal GBM cell survival in vitro, and increases mouse glioblastoma survival in vivo (tumor xenograft) [172]. In mesenchymal GBM, CCL5 inhibits apoptosis through the engagement of the unconventional receptor CD44. The hypothesis is that paracrine factors (secreted by the TME) important for low-grade glioma growth are produced in an autocrine way by high-grade tumors, creating regulatory circuits that maintain malignant glioma growth and survival [172].

CCL8 is a potent inhibitor of HIV-1 thanks to its high-affinity binding to the receptor CCR5 [173]. Zhang et al. [133] found that CCL8 secreted by TAMs induces invasion and stem-like traits of GBM cells, and CCR1 and CCR5 are the main receptors that mediate CCL8-induced biological behavior. CCL8 activates ERK1/2 phosphorylation in GBM cells, and blocking TAM secretion of CCL8 with anti-CCL8 neutralizing antibody decreases TAM-induced invasion of glioma cells [133].

Laudati et al. [131] found a relation between the chemokine-CCR5 system and the immunosuppressive polarization of microglia in GBM. Maraviroc prevented the occurrence of an M2 anti-inflammatory microglia state (ARG1+ and IL-10+). It counteracted the activity of glioblastoma-associated immunosuppressive M2-microglia, inducing their conversion to pro-inflammatory M1-microglia, and reduced microglia migration [131].

#### 3.2.8. Head and Neck Cancer and Squamous Cell Carcinoma of Tongue and Floor of the Mouth

Gonzalez et al. [49] evaluated the immunohistochemical expression of chemokine receptors in a retrospective cohort of 76 cases of head and neck squamous cell carcinoma, and found a positive correlation between CCR5 expression and lymph node metastasis, advanced clinical stage, poor tumor differentiation, recurrence, and shortened disease-free survival [49]. Both CCR5 and CCR7 were found to be markers of poor prognosis in patients with squamous cell carcinoma of tongue and floor of the mouth [174].

#### 3.2.9. Lung Cancer

CCL5 is involved in lung cancer cell (A549 cell line) migration by activating the PI3K/Akt/NF-kB pathway that increases the expression of integrin αvβ3 [56]. CCL5 was identified as a target of miR-147a that is down-regulated in non-small-cell lung cancer cell lines and tissues [134]. By modulating CCL5 expression, miR-147 inhibits tumor cell growth, migration, and invasion in vitro. Accordingly, miR-147-overexpressing cancer cells injected subcutaneously into mice inhibited tumor xenograft growth, showing reduced CCL5 expression, invasion, and decreased metastasis formation [134]. CCL5 is modulated at the epigenetic level by EZH2 (enhancer of zeste homolog 2) gene [135]. In vitro, EZH2 knockdown in lung cancer cells decreased CCL5 expression, resulting in reduced chemotaxis of macrophages. In vivo, EZH2 knockdown reduced macrophage infiltration, leading to decreased tumor growth and metastasis formation [135]. CCR5 expression in lung cancer was associated with immune cell infiltration and better overall survival [175].

#### 3.2.10. Melanoma

CCR5 is more expressed in melanoma cells than in normal melanocytes and is positively associated with tumor progression in patients [50]. In vivo studies demonstrated that CCR5 absence in B16/F10 or A375 melanoma cell lines decreases primary tumor growth and lung metastasis, while CCR5 overexpression enhances both growth and metastasis formation. CCR5 is involved in proliferation and migration of melanoma cells in vitro and supports the mesenchymal phenotype of metastatic melanoma cells by increasing TGF-β1, which in turn induces EMT and migration via PI3K/AKT/GSK3β signaling [50].

Malignant melanoma is characterized by the accumulation of MDSCs [136], and CCR5 is involved in both recruitment and activation of MDSCs in melanoma lesions [137]. In transgenic mouse melanoma models, the accumulation of CCR5+ MDSCs in primary tumors and metastatic lymph nodes correlated with tumor progression. CCR5 expression on MDSCs correlates with high levels of CCR5 ligands in melanoma TME and is induced by CCR5 ligands together with IL-6, granulocyte-macrophage colony-stimulating factor (GM-CSF), and other inflammatory factors. CCR5+ MDSCs infiltrating melanoma lesions are more immunosuppressive than CCR5− MDCSs. Neutralization of CCR5 ligands increased the survival of tumor-bearing mice and decreased both the migration and immunosuppressive potential of MDSCs. Melanoma patients had higher levels of circulating CCR5+ MDSCs than healthy donors, and CCR5 ligands were higher in tumors than in peripheral blood. The hypothesis is that CCR5 ligands are involved not only in MDSC recruitment but also in their immunosuppressive polarization [136,137]. Therefore, given that melanoma is highly immunogenic and also immune evasive, inhibiting the CCL5/CCR5 axis with maraviroc was recently proposed as the next step in melanoma immunotherapy [176].

#### 3.2.11. Osteosarcoma

Osteosarcomas are derived from primitive mesenchymal cells which produce osteoid and/or immature bone. High CCL5 levels positively correlate with metastasis and poor prognosis in osteosarcoma [177]. Osteosarcoma cells express CCR5 [57] and its engagement by CCL5 leads to the activations of αvβ3 integrin and the migration of osteosarcoma cells by activating NF-kB. Thus, CCL5 may serve as a biomarker for osteosarcoma prognosis, and may be a potential molecular target for osteosarcoma therapy.

#### 3.2.12. Ovarian Cancer

In ovarian cancer, both CCL5 and CCR5 are mainly expressed by CD133+ ovarian carcinoma stem-like cells [59], which are able to self-renew, induce tumor relapse, and form metastases. In CD133+ ovarian carcinoma stem-like cells selected from ovarian carcinoma cell lines and primary tissues, CCL5 and its receptors CCR5, CCR1, and CCR3, were up-regulated [59]. Blocking CCL5 or the corresponding receptors decreased the invasive capability of ovarian carcinoma cells, suggesting that the CCL5/CCR5 axis is important to the metastatic property of ovarian carcinoma stem-like cells [59]. CCL5 mediates invasiveness by activating NF-kB and increasing the secretion of MMP-9, both up-regulated in ovarian carcinoma stem-like cells and also in primary tumors [59].

Autocrine CCL5 signaling, by activating NF-kB and STAT3, induces ovarian carcinoma stem-like cells to differentiate into endothelial cells and enhances angiogenesis [141]. CCL5 secreted by CD133+ ovarian carcinoma stem-like cells increases the invasive and metastatic properties of non-ovarian carcinoma stem-like cells (CD133–) and induces an EMT-like process [140]. CCL5 and CCR1/CCR3/CCR5 expression correlates with tumor invasiveness, since none of these molecules are expressed in ovarian precancerous tissues but all are expressed in primary cancer and metastatic tissues [140].

Peritoneal dissemination is the most common mechanism of disease progression in ovarian cancer [178]. It is associated with the development of malignant ascites, which is a major cause of chemoresistance and recurrence. Ascites consists of tumor cells, mesothelial cells, fibroblasts, macrophages, leukocytes, and red blood cells in a complex protein-rich fluid. Ascites of ovarian carcinoma contains CCL3, CCL4, and CCL5 at levels higher than in patient plasma [179]. CCL5 levels correlate with the presence of CD3+ T cells, and among the CD3+ population, CD4+ T cells express more CCR5 and are predominant respect to CD8+ T cells [179]. Serum CCL5 levels are higher in women with ovarian cancer than in women with benign ovarian cysts, correlating with the extent of disease. Higher concentrations of CCL5 are also observed in patients with residual tumor mass than without it, confirming that CCL5 plays a role in ovarian carcinoma progression and suggesting that serum CCL5 levels are a potential diagnostic and prognostic marker [180].

CCR5 ligands are involved in TME formation. Tregs from ovarian carcinoma patients express high levels of CCR5, and treatment with conditioned medium from ovarian carcinoma stem-like cells increases their expression of IL-10 and MMP-9 [181].

Coculture of ovarian carcinoma cells with fibroblasts modifies the expression of miRNAs [182]. Cross-talk between ovarian carcinoma and fibroblasts decreases miR-31 and miR-214, and increases miR-155 expression, reprogramming normal fibroblasts into tumor-promoting CAFs (miR-CAFs). miR-CAFs gain enhanced migration, and ovarian carcinoma cells increase colony formation and invasion potential. Among the genes that were deregulated in miR-CAFs and also in patient-derived CAFs, CCL5 was the most up-regulated. Indeed, CCL5 is a direct target of miR-214. In accordance, anti-CCL5 antibody decreased tumor growth and the invasive property induced by miR-CAF co-injected in an orthotopic ovarian cancer mouse model, confirming CCL5 is a tumor-promoting factor [139].

In ovarian carcinoma cells, drug resistance can be intrinsic or promoted by the TME [183]. Conditioned medium from CAFs isolated from patients with high-grade serous ovarian cancer induces cisplatin resistance in C13 and SKOV3 ovarian carcinoma cell lines [62]. Cisplatin was found to up-regulate CAF-secreted CCL5. CCL5 induced ovarian carcinoma cell drug resistance by activating pSTAT3 and PI3K/pAKT pathways. Co-injection of tumor cells and CAFs increased CCL5 expression, cisplatin resistance and ovarian carcinoma cell proliferation in tumor xenografts [62]. Consistently, in ovarian carcinoma tissues, tumor cells and stromal cells increased CCL5 after cisplatin treatment, and cisplatin-resistant patients had high CCL5 levels that correlated with cancer stage [62].

MSCs protect ovarian carcinoma cells from chemotherapy [184] by secreting CCL5 and CCL2, which induce the release of IL-6 by ovarian carcinoma cells. Engagement of IL-6 in ovarian carcinoma cells phosphorylates PYK2, resulting in chemoresistance to carboplatin and taxanes. The anti-human IL-6R monoclonal antibody tocilizumab, in association with chemotherapy, reduced peritoneal carcinosis index compared to chemotherapy alone, in mice injected with ovarian carcinoma cells and MSCs [184].

CCR5 antagonist anibamine and its analogues inhibited the CCL5-induced proliferation and the intracellular Ca^2+^ flux of OVCAR-3 ovarian cancer cells, with no significant toxicity in NIH/3T3 fibroblasts [138]. Cordycepin, by down-regulating CCL5, prevented constitutively Akt-mediated NF-κB transcription factor activation in cells, leading to decreased migration and apoptosis induction [185].

#### 3.2.13. Pancreatic Cancer

Singh et al. [51] found high epithelial cells staining of CCR5 and CCL5 in metastatic tissues compared to non-neoplastic. Pancreatic cancer cell lines express high levels of CCR5, and CCL5 induced proliferation and increased the invasive potential of cancer cells. Maraviroc reduced both proliferation and invasion induced by CCR5 engagement [51]. Pancreatic ductal adenocarcinoma (PDAC) is the most prevalent neoplastic disease of the pancreas, accounting for more than 90% of all pancreatic malignancies. Maraviroc inhibits the proliferation of Suit2-007 and MIA-PaCa-2 human PDAC-derived cell lines, induces caspase activation, Bax increase, and inhibits cell cycle progression [142]. In vivo (rat xenograft model), maraviroc reduced PDAC liver metastasis [142]. These results also suggest that maraviroc could be a promising treatment for PDAC patients with liver metastases.

#### 3.2.14. Pituitary Tumors

Pituitary tumors with mutated-aryl hydrocarbon receptor interacting protein have an aggressive phenotype [186]. This tumor is characterized by an intratumoral accumulation of macrophages and high levels of CCL5 expression compared to sporadic adenomas or normal pituitary [143]. Macrophages secrete molecules that induce EMT and enhance the invasiveness of mutated cancer cells. Conditioned medium from the pituitary GH3 tumor cell line increased macrophage migration, which is inhibited by maraviroc [143].

#### 3.2.15. Prostate Cancer

The CCL5/CCR5 axis is involved in prostate cancer progression: both molecules are expressed in human prostate cancer cell lines, primary cultures of prostatic adenocarcinoma cells and prostate cancer tissues [52]. High levels of CCL5 were found in blood samples and were associated with poor prognosis [187]. Prostate specific membrane antigen (PSMA) is a diagnostic biomarker because its expression is elevated in high-grade prostate cancer, in metastatic prostate cancer, and in androgen-insensitive prostate carcinoma [188]. PSMA activation induces the p38 and ERK1/2 MAPKs cascade leading to NF-kB activation, and increases both CCL5 and IL-6 expression [189]. CCL5 increases tumor growth by activating STAT5 and cyclin D1 pathways, enhanced tumor invasion and synergizes with IL-6 [189]. TAK-779 inhibits both CCL5-induced proliferation and invasion of tumor cells [52].

CCR5 antagonist anibamine inhibited prostate cancer cell line growth with and without CCL5 stimulation in vitro, and decreased prostate cancer xenograft growth [81]. CCR5 is a target of miR-455-5p [145]. The down-regulation of CCR5 by miR-455-5p overexpression inhibited prostate cancer cell growth and induced apoptosis [145]. In human prostate cancer tissues, miR-455-5p levels are significantly lower than in healthy tissues, suggesting miR-455-5p as a possible prognostic and diagnostic biomarker [145].

Paclitaxel-resistant PC3 prostate cancer cells show elevated expression of CCR1. Its engagement through CCL5 ligand increases tumor cell invasive properties by enhancing MMPs-2 and MMPs-9 production via ERK and Rac [60].

The interaction of prostate cancer cells with MSCs increases CCL5 secretion in both cell types [190] and conditioned medium from prostate cancer cells increases CCL5 secretion by MSCs [191]. CCL5 secreted by prostate cancer cells induces the migration of MSCs [191].

Increased CCL5 levels activate hypoxia inducible factor 2α and down-regulate tumor cell androgen receptor signals [192]. Suppression of androgen receptor signaling augments the cancer stem cell population with high invasive properties [192].

Endothelial cells, by secreting CCL5, also inhibit androgen receptor signaling and promote tumor cell invasion [144]. Androgen receptor suppression induces autophagy, which further promotes cancer cell motility by increasing the disassembling of paxillin at focal adhesions [144]. Maraviroc, in combination with chloroquine (an autophagy inhibitor), reduced metastasis in prostate cancer orthotopic mouse models and improved overall survival [144].

Recently, the immunofluorescence analysis of primary tumor and metastatic lymph node tissues showed a high colocalization of CCL5 with TAMs [187]. CCL5 secreted by TAMs increases prostate cancer stem cell expansion and tumor cell invasion via activating β-catenin/STAT3 signaling [187].

#### 3.2.16. Thyroid Carcinoma

Platelets, by secreting CCR5 ligands [193], support tumor cell progression [194,195]. In anaplastic thyroid carcinoma patients, platelets are implicated in metastasis, and high platelet counts in peripheral blood are an adverse prognostic factor [196,197]. Activated platelets, by secreting CCL3, engage CCR5 expressed by anaplastic thyroid carcinoma, activate NF-kB, up-regulate MMP1, and enhance migratory and invasive properties of cancer cells [197].

## 4. Counteracting the CCL5/CCR5 Axis: Clinical Applications

The TME has crucial roles in tumor initiation, progression, and metastasis [198]. As a consequence, to improve patient outcome, new therapeutic strategies should kill tumor cells and counter the formation of a tumor-promoting TME. In this context, numerous studies in different tumor types have demonstrated the CCL5/CCR5 axis to be involved in tumor growth, metastasis, and the building of an immunosuppressive tumor-promoting TME.

Many preclinical studies have concluded that counteracting CCR5 engagement should be an effective treatment against tumors whose progression depends on CCL5/CCR5 interactions, providing the basis for clinical trials. Moreover, targeting CCR5 and/or CCL5 may be effective in combination with immune checkpoint inhibitors or DNA damaging agents.

### 4.1. Inhibition of CCR5/CCL5 Interactions in Clinical Trials

The CCR5/CCL5 axis has been studied in many preclinical studies by CCL5 silencing or neutralization and by using CCR5 antagonists. These studies support the translation of CCR5 antagonists, like maraviroc, from bench to bedside, i.e., from laboratory experiments to clinical trials (Table 3).

CCR5 antagonists, normally used to block HIV entry into T cells and macrophages in AIDS patients and to protect against graft-versus-host disease in transplant recipients, were recently employed for cancer treatment. Clinical trials with the CCR5 antagonists maraviroc, leronlimab, vicriviroc and BMS-813160 [17] in triple-negative breast cancer, colorectal cancer, Kaposi’s sarcoma, and advanced pancreatic ductal carcinoma are completed, ongoing or recruiting (Table 3). CCR5 antagonists are being tested in combination with the checkpoint inhibitors pembrolizumab and nivolumab, humanized monoclonal anti-PD-1 antibodies that block PD-1 on T cells, preventing their inactivation by PD-L1 expressed by cancer cells, TAMs, and CAFs.

### 4.2. Pharmacological Inhibition of CCL5 Secretion by Cancer Cells and the TME

Another strategy to slow cancer progression at the CCL5/CCR5 axis level is inhibition of CCL5 secretion, which can also be achieved by drugs that do not have CCL5 as their target. For instance, trabectedin [199] and auranofin decreased the secretion of CCL5 by liposarcoma and cHL cells, respectively. Zoledronic acid affects the secretion of CCL5 and IL-6 by MSCs [200], decreasing their effects on breast cancer cells [10]. The tyrosine kinase inhibitor gefitinib inhibits epidermal growth factor receptor (EGFR) activation on MSCs by conditioned medium from PC3 prostate cancer cells (bone metastasis of prostate cancer), leading to decreased secretion of CCL5 [191]. In addition, zoledronic acid blocks the interaction between breast cancer cells and Tregs [201]. Emodin, a natural anthraquinone derivative with tyrosine kinase inhibitory activity, was found to up-regulate the expression of E-cadherin and inhibit the expression of vimentin, β-catenin, and snail by decreasing the expression of CCL5 in adipose tissue of cancer patients [202]. Emodin inhibits CCL5 secretion from adipocytes and counteracts EMT in triple-negative breast cancer cells. Moreover, it inhibits cancer growth and metastasis to the lung and liver, which indicates a role of emodin in preventing the metastasis of triple-negative breast cancer [202].

## 5. Conclusions

Many preclinical studies in different cancer models have demonstrated the protumorigenic role of the CCL5/CCR5 axis, which is involved not only in tumor growth and metastasis but also in the formation of an immunosuppressive and protective TME. Accordingly, the overexpression of CCL5 or CCR5 by cancer cells or tumor tissues has been found to correlate with poor prognosis and CCR5 expression by cancer cells to associate with a decreased activity of DNA damaging agents.

This evidence provides the basis for the clinical assessment in cancer treatment of the repurposed drug maraviroc, as a single agent or in combination with immune checkpoint inhibitors or DNA damaging agents to potentiate their cytotoxic activity, and of drugs capable to decrease CCL5 levels in tumor tissues.

## Figures and Tables

**Table 1:** Effects of CCL5/CCR5 axis disruption in hematological malignancies.

Tumor Type	Molecules	Related Studies/Effects	Ref.
Acute lymphoblastic leukemia	Maraviroc	Induced tumor cell apoptosis in vitro, and decreased tumor xenograft growth.	[39]
Acute myeloid leukemia	Maraviroc	Inhibited T-reg accumulation and increased antileukemic effects of CD8+ T cells in tumor xenografts.	[85]
Hodgkin lymphoma	Anti-CCL5 antibody	Inhibited autocrine tumor growth; reduced tumor cell recruitment of CD4+T cells, eosinophils, monocytes, and MSCs; Inhibited tumor cell clonogenic growth induced by CCL5 secreted by tumor-educated MSCs.	[38,40]
	Maraviroc	Inhibited: clonogenic growth of tumor cells; monocyte migration induced by tumor-educated MSCs; heterospheroids formation and viability; tumor xenograft growth, and monocyte accumulation in tumor tissues. Enhanced doxorubicin and brentuximab cytotoxic activity.	[38]
Multiple myeloma	TAK779	Inhibited bone marrow homing of tumor cells, osteoclastogenesis and osteoclastic resorption in tumor xenografts.	[41]
	Anti-CCR5 antibody	Inhibited osteoclast formation and myeloma cell adhesion to stromal cells.	[86]

C-C chemokine ligand 5, CCL5; C-C chemokine receptor type 5, CCR5; mesenchymal stem cell, MSC; regulatory T cell, Treg.

**Table 2:** Effects of disrupting the CCL5/CCR5 axis in solid tumors.

Tumor	Treatment	Effects	Reference
Breast cancer	Maraviroc	Decreased Treg migration, reduced lung metastasis in mouse models	[117]
		Inhibited angiogenesis, reduced metastasis and improved survival in mouse models	[118]
		Reduced immune-suppressive myeloid cells, increased antitumor immunity and reduced tumor growth in mouse models (in presence of CCL5-targeting siRNA)	[78]
		Reduced mice tumor growth and thoracic metastasis (in presence of anti-IL-6R)	[119]
		Reduced metastasis in mouse models Enhanced cell killing by doxorubicin in vitro	[43]
		Reduced resistance to trastuzumab due to CCL5 overexpression	[120]
	Anti-CCL5 antibodies	Counteracted the ability of MSCs to induce metastasis in tumor xenografts	[10]
		Inhibited collagen VI production by fibroblasts in response to the paracrine coculture of adipose-derived stem cells and tumor cells	[121]
Breast phyllodes	Maraviroc	Inhibited monocyte recruitment and tumor growth in patient-derived mouse xenografts	[122]
Cholangio- carcinoma	Anti-CCL5 antibody, maraviroc	Decreased cell invasion and CCR5 expression induced by MSCs	[123]
Colon cancer	Maraviroc	Decreased CAF and tumor xenograft growth	[124]
		Repolarized immunosuppressive TAMs towards an anti-tumoral M1-like state in organoids from patients with metastatic CRC. Generated objective clinical responses in CRC patients (MARACON-001 phase I trial)	[44]
		Reduced MSC-induced tumor xenograft growth in vivo	[125]
	Anti-CCL5 antibodies	Decreased in vitro growth and migration of tumor cells, xenograft growth, liver metastases, sensitized to PDGFRβ therapy	[126]
		Inhibited the effects of macrophage CCL5: PDL-1 stabilization and inhibition of tumor killing by T cells	[127]
	RNAi-CCL5 silencing	Decreased tumor growth in immunocompetent syngeneic mice and reduced Treg infiltration	[4]
	CCL5 knockout	Decreased tumor growth and metastasis facilitating CD8+ T cell accumulation in the tumor site in CRC xenografts. Decreased resistance to anti-PD-1 therapy	[128]
Esophageal carcinoma	Maraviroc, siRNA-CCL5 silencing	Blocked tumor cell migration and invasion in vitro. Decreased cell growth rate, migration and invasion, and induced apoptosis in vitro	[46]
Gastric cancer	Anti-CCL5 antibodies	Decreased tumor invasion induced by CCL5 secreted by PBMCs educated by tumor cells	[129]
		Decreased in vivo growth of tumor cells coinjected with PBMCs	[130]
Glioblastoma	Maraviroc, siRNA-CCR5 silencing	Induced M1 polarization of microglia and reduced migration	[131]
		Inhibited tumor growth, invasion, and xenograft growth	[132]
	Anti-CCL8 antibody	Reduced invasion and stemness of tumor cells	[133]
Lung cancer	Anti-CCL5 antibody	Decreased tumor cell migration, invasion, and colony formation; inhibited growth and metastasis in xenografts	[134]
	shRNA-EZH2 silencing siRNA-CCL5 silencing	Reduced tumor cell migration and invasion, suppressed metastasis and macrophage infiltration in xenografts	[135]
Melanoma	mCCR5-Ig	Increased survival of tumor-bearing mice and decreased migration and immunosuppressive potential of MDSCs	[136,137]
Ovarian cancer	Anibamine	Inhibited tumor cell growth and intracellular Ca^2+^ flux	[138]
	shRNA-CCL5 silencing, anti-CCR5 antibody	Inhibited the invasive capacity of CSLCs, inhibited invasion of ovarian cancer stem-like cells in vitro and in tumor xenografts	[59]
	Anti-CCL5 antibody	Decreased tumor growth and invasion induced by CAFs	[139]
		Reduced epithelial-mesenchymal transition-like process of non-stem ovarian cancer cells, metastasis in xenografts	[140]
	Anti-CCL5 antibody, shRNA-CCL5 silencing	Inhibited endothelial cell differentiation and tube formation of cancer stem-like cells in vitro and in xenografts	[141]
Pancreatic cancer	Maraviroc	Decreased tumor cell growth and invasion	[51]
		Decreased tumor cell Inhibited, induced apoptosis in vitro, reduced liver metastasis from xenografts	[142]
Pituitary tumor	Maraviroc	Decreased macrophage migration induced by tumor cells	[143]
Prostate cancer	Anibamine	Reduced tumor xenograft growth	[82]
	Maraviroc	Reduced metastasis and improved overall survival (in presence of chloroquine)	[144]
	miR-455-5p	Inducted apoptosis, suppressed cellular proliferation, and tumor xenograft growth	[145]

Cancer-associated fibroblast, CAF; C-C chemokine ligand 5, CCL5; C-C chemokine receptor type 5, CCR5; colorectal cancer, CRC; enhancer of zeste homolog 2, EZH2; mCCR5-Ig, fusion protein that neutralizes all three CCR5 ligands; mesenchymal stem cells, MSC; myeloid-derived suppressor cell, MDSC; platelet-derived growth factor receptor, PDGFR; programmed death ligand 1, PD-L1; peripheral blood mononuclear cell, PBMC; shRNA short hairpin RNA; small interfering RNA, siRNA; tumor-associated macrophage, TAM; regulatory T cell, Treg.

**Table 3:** Clinical trials with CCR5 inhibitors.

Treatment	Tumor	Name (Recruitment Status)	ClinicalTrials.gov Identifier
Leronlimab + carboplatin	Triple-negative breast cancer	A Phase Ib/II Study of Leronlimab (PRO 140) Combined With Carboplatin in Patients With CCR5+ Metastatic Triple Negative Breast Cancer (recruiting)	NCT03838367
Leronlimab	Triple-negative breast cancer	A Compassionate Use Study of Leronlimab (PRO 140) in Combination With Treatment of Physician’s Choice in Patients With CCR5+ Metastatic Triple Negative Breast Cancer (available)	NCT04313075
Maraviroc	Colorectal cancer	Treatment of Advanced Colorectal Cancer Patients With Hepatic Liver Metastases Using the CCR5-Antagonist Maraviroc (Phase I Maracon Trial) (completed)	NCT01736813
Maraviroc + pembrolizumab	Colorectal cancer	A Phase I Trial of Combined PD-1 Inhibition (Pembrolizumab) and CCR5 Inhibition (Maraviroc) for the Treatment of Refractory Microsatellite Stable (MSS) Metastatic Colorectal Cancer (PICCASSO) (completed March 2020)	NCT03274804
Vicriviroc + pembrolizumab	Microsatellite stable colorectal cancer	A Phase 2 Trial to Evaluate the safety and Efficacy of Vicriviroc (MK-7690) in Combination With Pembrolizumab (MK-3475) in Participants With Advanced/Metastatic Microsatellite Stable Colorectal Cancer (active, not recruiting)	NCT03631407
Maraviroc	Kaposi’s sarcoma	Effects of Maraviroc on HIV-related Kaposi’s Sarcoma (completed)	NCT01276236
BMS-813160 + nivolumab	Advanced pancreatic ductal adenocarcinoma	A Phase I/II Trial of Combination Immunotherapy With Nivolumab and a CCR2/CCR5 Dual Antagonist (BMS-813160) With or Without GVAX Following Chemotherapy and Radiotherapy for Locally Advanced Pancreatic Ductal Adenocarcinomas (recruiting)	NCT03767582

Leronlimab, humanized monoclonal anti-CCR5 antibody; maraviroc, CCR5 antagonist; pembrolizumab, humanized monoclonal anti-PD-1 antibody; vicriviroc, CCR5 antagonist; BMS-813160, dual antagonist of CCR2/CCR5; GVAX pancreas vaccine (granulocyte-macrophage colony-stimulating factor-secreting allogeneic pancreatic tumor cells); nivolumab, anti PD-1; GVAX, cancer vaccine.
